# The effects of the flipped classroom in teaching evidence based nursing: A quasi-experimental study

**DOI:** 10.1371/journal.pone.0210606

**Published:** 2019-01-15

**Authors:** Tsung-Lan Chu, Jeng Wang, Lynn Monrouxe, Yu-Chih Sung, Chen-li Kuo, Lun-Hui Ho, Yueh-E Lin

**Affiliations:** 1 Department of Quality Management, Administration Center, Chang Gung Medical Foundation, Taoyuan, Taiwan; 2 Department of Nursing, Chang Gung University of Science & Technology, Taoyuan, Taiwan; 3 Department of Nursing, Linkou Chang Gung Memorial Hospital, Taoyuan, Taiwan; 4 Chang Gung Medical Education Research Centre (CG-MERC), Chang Gung Memorial Hospital, Gueishan, Taiwan; 5 Department of Information Management, Chang Gung University, Taoyuan, Taiwan; 6 School of Nursing, National Taipei University of Nursing and Health Sciences, Taipei, Taiwan; Waseda University, JAPAN

## Abstract

**Background:**

Evidence-based nursing (EBN) has been an important training mechanism for improving the quality of clinical care. At present, the pedagogy focuses on the application of e-learning and team-based learning to enhance learners’ engagement and learning effectiveness.

**Objectives:**

This study applied the flipped classroom approach to conduct evidence-based nursing (EBN) teaching. The aim of this study is to elevate the learning effectiveness of the flipped classroom group to the traditional teaching group in terms of knowledge and self-efficacy in practice.

**Design:**

A pretest-posttest nonequivalent control group with a quasi-experimental quantitative design.

**Methods:**

The study recruited 151 nurses, of whom 75 were in the control group and 76 were in the experimental group. During the EBN course, the control group received training via traditional pedagogy while the experimental group engaged the flipped classroom approach. The learning effectiveness of EBN knowledge and self-efficacy in practice were evaluated across the three time points: pre-course, post-course, and one month after the course.

**Results:**

In both group the scores of the EBN knowledge and self-efficacy in practice improved after training. The scores of the experimental group increased significantly than in the control group. However, the scores declined in both groups one month after the course. Even so, the experimental group’s score of self-efficacy in practice was still higher than that of the control group.

**Conclusion:**

The implementation of the flipped classroom approach and team-based learning effectively enhanced the learners EBN knowledge accumulation and self-efficacy in practice. The research results can be used as an important reference for improving clinical nursing teaching quality.

## Introduction

### Evidence-based nursing (EBN)

Medical personnel is encouraged to make clinical decisions with the best evidence available to provide patients with appropriate nursing care strategies [1–3]. Evidence-based nursing (EBN) was devleoped on the basis of offering patient care with scientific methodolgy, which means systematic search of the literature and access to the best literature as the best evidence to support clinical decision making in clinical care [4]. It consists of seven steps, which were identified as step (0) Cultivate a spirit of inquiry, step (1) Ask an answerable question via the PICOT (Problem, Intervention, Comparison, Outcome, Time) format, step (2) Acquire the best available evidence according to the questions, step (3) Appraise the evidence for its validity, step (4) Apply the strategy to your patients, step (5) Audit the performance of above procedures and step (6) Disseminate EBP results [5–7].

Evidence-based nursing care has been of considerable concern for clinical continuing education [3, 8] and become one of the clinical education evaluation indexes in nursing. Applying EBN training enables nurses to consider valid research, professional experiences and patients’ preferences into clinical care management [3, 9], and improve nurses’ clinical care performance, self-efficacy, and internet search skills [10, 11].

Although EBN can improve nurses’ ability and performance of clinical care, the application of EBN into clinical is impeded by demographic factors such as educational level, age, working years [12], and personal factors such as lack of EBN skills, lack of organizational support [13, 14], and insufficient time. From the institutional perspective, the lack of earmarked fund [15], human resource shortage, unorganized supervision system, insufficient databases, and inadequate hardware have been shown to be obstacles for EBN implementation [16]. Furthermore, nurses form non-English speaking countries had an additional language factors affecting their implementation of EBN in appraising English research articles [17]. In Taiwan, nurses who lack of confidence in reviewing English literature show low motivation to conduct literature appraisal and presentation. Therefore, it urges for reformation of the EBN education[18].

Indeed, the present methods of delivery information to students are no longer applicable. In order to attract students’ interest, the pedagogy requires more active and diverse strategies. The flipped classroom teaching is to meet the above requirements and promote students’ engagement and critical thinking.

### The flipped classroom

The flipped classroom has been widely used in various countries and is recognized as an innovative and important teaching strategy in the field of higher education [19]. There is a call for education institutes to develop the flipped classroom method and improve the classroom atmospheres and learning attitude [20, 21]. This approach emphasize switching the educator’s instruction to the learner’s self-learning. It moves learning outside the classroom and allows students to preview new material, while leave classroom time for content-related activities and collaborative tasks [22–25]. The in-class activities can be held with several learning strategies, such as role playing, group problem solving, simulation, case studies, and feedback [26]. This innovative approach enables educators to offer one-on-one instruction and fosters student’s sense of responsibility for learning [27, 28]. The role of educator is converted from leader to coordinator, providing guidance and assistance [25,27,29]. Consequently, the flipped classroom model can improve teaching and enable learners to apply and integrate the taught contents and even learning to evaluate and create [30].

Flipped learning enhances learner engagement and academic outcomes [26, 31], however, its success varies by discipline and research measurement issues. There are still few issues on the evaluation of learning effectiveness of flipped classroom. Some of the previous researches lacked statistical data, significant intervention, and comparison group and needed further examination on flipped classroom strategy efficacy [18]. Also, recent studies found inconsistent intervention outcomes of applying the flipped classroom strategy [32]. Thus, multi-disciplinary collaborations have been recommended in order for the development of an effectiveness evaluation of the flipped classroom [33].

As technology advances and affects education, learning is no longer confined to traditional classroom and textbooks. The latest information and resources can be accessed via online learning without the limitations of time and space [28, 34]. Applying mobile technology to deliver information can break the time barriers and enable learners to access information in moments [35]. In addition, the application of mobile technology increases the communication and information exchange. Also, via the integration of knowledge management and e-learning, it creates a competitive advantage to foster learners’ learning. The information technology has also been used in medical promotion to enhance the effectiveness of clinical care [36, 37].

The flipped classroom model has attracted a great deal of interest in education [26]. However, limited research has discussed about the effectiveness of flipped classroom on evidence-based nursing. This study applied flipped classroom strategy to the Evidence-based nursing (EBN) course for clinical nursing staff training. The course was developed through a collaboration between clinical educators, nursing faculty and experts in computer science in order to enhance the learning effectiveness of evidence-based nursing (EBN) teaching.

## Aim

The aim of this study was: To compare the learning effectiveness of the experimental group who received courses via the flipped classroom teaching strategy to the control group who received courses via traditional classroom learning in terms of knowledge and self-efficacy in practice.

## Methods

### Design

A pretest-posttest nonequivalent control group with a quasi-experimental quantitative design was used in this study. The study had been approved by Chang Gung Medical Foundation Institutional Review Board (IRB No. 106-0828C).

### Participants

**Fig 1:**
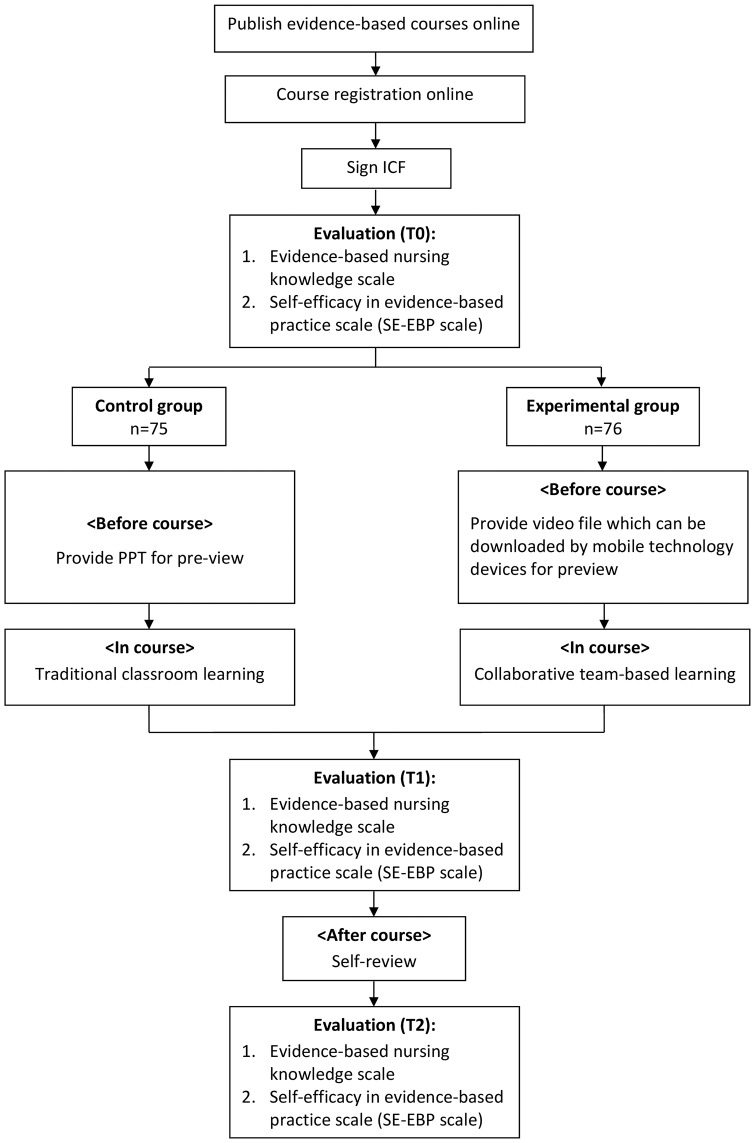
Research procedure.

With convenience sampling, the participants were recruited from a medical center in northern Taiwan. Nursing staff who registered for the EBN in-service training course were invited to the study. Participation inclusion criteria were (1) employed nursing staff, (2) aged ≥ 20 years, and (3) willing to sign a consent form to participate in the study. A total of 151 nurses were enrolled in this study. In order to prevent subject pool contamination, the first 75 nurses were assigned to the control group and the following 76 nurses were assigned to the experimental group (Fig 1)

### Intervention

Under the same course content, the control group received EBN training via traditional classroom-based approach, while the experimental group engaged in the flipped classroom approach. Among the seven steps of EBP, step zero (Cultivate a spirit of inquiry) was integrated into step one, and the step six (disseminate) can only be reached after the completion of EPP application and evaluation, the course therefore mainly focused on EBN 5As steps “Ask, Acquire, Appraise, Apply and Audit” [38]. The whole process mentioned above was under the same instructor’s coordination, whom was an EBP instructor qualified by Taiwan Evidence-Based Medicine Association.

The EBN course consists of a three-hour content-based section of the EBN 5As, and a two-hour practice in relation to EBN stage 3 (appraising research articles). The control group received three-hour traditional classroom-based instruction on the EBN 5As, followed by two-hour practice. The experimental group were asked to pre-read two multimedia training materials about the EBN 5As and spent the three-hour content-based section on practice, discussion and clarification on the EBN 5As, followed by a two-hour practice on appraising research articles. The two-hour practice was conducted in both the control and experimental groups, they were both assigned into subgroups and applied team-based and discussion into research articles appraisal. They were both freely to express their opinions on the issues discussed, and they were encouraged to share their thoughts when the opinions were inconsistent. The learning outcomes of the nurses’ EBN knowledge and self-efficacy in practice were evaluated at three time points: before the course (T0), after the course (T1), one month after the course (T2). T0 was for establishing the baseline scores, and T2 was for testing the long-term learning effect (Fig 1)

### Instruments

The study used a basic information questionnaire, an EBN knowledge scale, and the self-efficacy in evidence-based practice (SE-EBP) scale. The details of the questionnaire and the scales were as follows:

**Basic information questionnaire**: including gender, age, marital status, education, working years, work unit, and experiences of EBN course or practice.

**EBN knowledge scale**: The scale was developed based on the 5A steps of EBN. Three clinical EBN experts qualified by the Taiwan Evidence-Based Medicine Association (TEBMA) were invited to modify the scale into 10 single choice questions. The content validity index (CVI) value was 0.99 and the value of internal consistency (Cronbach’s α) is 0.76. Each item was accounted for 10 points and the range of score was from 0 to 100.

**Mandarin version of the SE-EBP scale**: The original version was developed by Chang and Crowe in 2011. This study used a translated version [38]. The content of the scale evaluated participants’ self-efficacy in the 5A steps of EBN. There were 28 questions in the scale. Three evidence-based experts qualified by TEBMA were invited to modify the scale and reduce the number of questions from 28 to 12, with the CVI value of 0.97and the internal consistency (Cronbach’s α) of 0.97. The modified version was a 10-point Likert scale and included 12 items. This score was calculated on a scale of 12–120 in 1-point increments, with 120 being the maximum and 12 being the minimum.

### Data analysis

The demographical data were presented as number and proportion for categorical variables and as mean and standard deviation (SD) for continuous variables. The outcome of EBN knowledge and self-efficacy were compared using independent t-test. Generalized Estimating Equation (GEE) was used to further identify the effectiveness of knowledge and self-efficacy in practice between the two groups over time. The data analyses were conducted using SPSS 17.0.

## Results

### Participant characteristics

The demographic data for the control group and the experimental group were examined via chi-square and two-tailed t-tests. The results showed that there were no differences between the two groups among all the variables. Both groups were with similar distribution in age, educational level, working ward, gender, marital status, rank, working years, and experiences of evidence-based course and practice (Table 1). The majority of participants were female and had bachelor degree. More than half of them were single and worked in general wards. Regarding the rank and working years, the participants were evenly distributed across each category. Although the experimental group participants had taken evidence-based courses previously, most of them lacked evidence-based practice.

**Table 1:** Demographic data analysis.

Variable	Group				*t*/x^2^	*P* value (2-tail)
	Control group (n = 75)		Experimental group (n = 76)			
	Mean±SD	n(%)	Mean±SD	n(%)		
**Age (years)**	33.61±8.498		35.20±9.120		1.104	.271
**Educational level**					1.789	.409
College		6(8.00%)		6(7.89%)		
University		64(85.33%)		60(78.95%)		
Graduate school		5(6.67%)		10(13.16%)		
**Ward**					.665	.717
General		40(53.33%)		40(52.63%)		
Emergency		29(38.67%)		27(35.53%)		
Others		6(8.00%)		9(11.84%)		
**Gender**					.327	.568
Male		1(1.33%)		2(2.63%)		
Female		74(98.67)		74(97.37%)		
**Marital status**					2.548	.280
Single		49(65.33%)		45(59.21%)		
Married		26(34.67%)		31(40.79%)		
**Rank**					2.084	.353
Training ~ N1		18(24.00%)		16(21.05%)		
N2 ~ N3		22(29.33%)		16(21.05%)		
N4		35(46.67%)		44(57.89%)		
**Working years**					4.758	.313
0 ~ 5yr		20(26.67%)		21(27.63%)		
6 ~ 10yr		17(22.67%)		8(10.53%)		
11 ~ 15yr		14(18.67%)		14(18.42%)		
16 ~ 20yr		10(13.33%)		15(19.74%)		
21yr and above		14(18.67%)		18(23.68%)		
**Evidence-based course experience**					1.508	.219
Yes		41(54.67%)		49(64.47%)		
No		34(45.33%)		27(35.53%)		
**Evidence-based practice experience**					1.359	.507
Yes		17(22.67%)		14(18.42%)		
No		58(77.33%)		61(80.26%)		

### Comparison of EBN knowledge and self-efficacy in practice

The scores of evidence-based knowledge and self-efficacy in practice for the control and experimental groups were compared across 3 time periods (T0-T2). T0 scores showed no difference between the two groups (Knowledge *p* = 0.52; Self-efficacy *p =* 0.786). T1 scores revealed that all the participants presented better performance, with the experimental group had significantly higher scores than the control group (knowledge *p =* 0.015; self-efficacy *p =* 0.011). However, T2 scores showed no significant differences in both groups (knowledge *p* = 0.893; self-efficacy *p* = 0.097) (Table 2).

**Table 2:** Comparison of evidence-based nursing knowledge and self-efficacy in practice between two groups.

Variable	Group		*t* value	*P* value (2-tail)
	Control group (n = 75) Mean±SD	Experimental group (n = 76) Mean±SD		
**Knowledge**				
Pretest (T0)	65.33±18.55	59.21±18.85	1.96	.520
Posttest (T1)	75.07±14.55	80.92±14.62	2.47	.015*
Posttest2 (T2)	74.89±17.68	74.46±4.76	.13	.893
**Self-efficacy**				
Pretest (T0)	63.61±16.39	62.76±21.66	.27	.786
Posttest (T1)	82.15±17.52	89.03±15.19	2.58	.011*
Posttest2 (T2)	81.32±17.80	86.86±15.72	1.68	.097

*P<0.05;

**P<0.01

### The improvement differences of learning effectiveness in EBN knowledge and self-efficacy in practice between the two groups over time

With regard to the individual improvement difference of learning effectiveness, generalized estimating equation (GEE) was applied to investigate individual improvement difference between groups. The average rank of all participants’ knowledge in posttest (T1) and posttest 2 (T2) was higher than the rank in pretest with the difference of 9.73 and 9.66 respectively, which reached the statistical significance. In terms of the learning effect between groups, the posttest-pretest knowledge difference of the experimental group was higher than that of the control group, with the difference of 11.98 (*p* < 0.001). However, the posttest 2-pretest knowledge differences between the two groups failed to achieve statistical significance. All the score differences of self-efficacy in practice, whether they are the average of all participants or group differences, showed a significant difference across the groups (Table 3).

**Table 3:** The learning effectiveness in evidence-based knowledge and self-efficacy in practice between two groups over times.

Variable	GEE (Interaction between time and groups)					
	*β*	SE	95%CI		Wald *X*^*2*^	*P*-value
			Lower	Upper		
**Knowledge**						
Group (Experimental group vs. Control group)	-6.12	3.11	-12.21	-.04	3.89	.049*
Learning effect						
Posttest vs. Pretest	9.73	1.86	6.09	13.37	27.48	<.001***
Posttest2 vs. Pretest	9.66	2.51	4.74	14.57	14.84	<.001***
Group* Learning effect						
Posttest vs. Pretest	11.98	2.58	6.91	17.04	21.47	<.001***
Posttest2 vs. Pretest	4.74	3.34	-1.82	11.29	2.01	.157
**Self-efficacy**						
Group (Experimental group vs. Control group)	-0.85	3.10	-6.93	5.23	.08	.784
Discrepancy						
Posttest vs. Pretest	18.53	1.71	15.18	21.89	117.43	<.001***
Posttest2 vs. Pretest	16.92	2.07	12.85	20.98	66.56	<.001***
Group* Discrepancy						
Posttest vs. Pretest	7.73	3.22	1.43	14.03	5.78	.016*
Posttest2 vs. Pretest	6.03	3.01	.13	11.92	4.02	.045*

*P<0.05;

**P<0.01;

***P<0.001

## Discussion

In our study, we found that EBN knowledge and self-efficacy were significantly elevated in both groups following the EBN courses, with the scores of the experimental group being significantly higher than those of the control group. In terms of EBN knowledge, the results therefore indicate that the teaching strategy and course design applied are effective for knowledge acquisition and self-efficacy.

However, the significant difference between flipped classroom and traditional lecture groups disappeared at one month after the course(T2). The scores of both knowledge and self-efficacy dropped from T1 to T2. The score of experimental group dropped from 80.94 to 74.46 in knowledge and from 89.03 to 86.86 in self-efficacy (Table 1), whereas the score of control group dropped from 75.07 to 74.89 in knowledge and from 82.15 to 81.32 in self-efficacy. The drop might be due to the length of the course, with short-term interventions limiting the efficacy of the teaching method [39]. The course we ran was based solely on a 5-hour training and thus is insufficient for predicting long-term performance (such as the outcomes after one month). Although the EBN course applied flipped classroom teaching and team-based learning in this study, the whole course was still focused on information delivery rather than providing nurses with virtual simulations to practice and apply the learned concepts in a stimulating context [40]. That could also explain the short-lasting impact of flipped classroom model on EBN knowledge accumulation. One way of overcoming this issue is to extend the intervention period to improve the long-term impacts of flipped classroom on EBN knowledge accumulation and practice performance.

In terms of the self-efficacy in EBN practice which has not been widely discussed, the experimental group’s scores of self-efficacy performed better than the control group in all the evaluation points after receiving the course. In flipped classroom teaching, students have more opportunities to think critically [26]. It is evident in the literature that the self-confidence can be increased via flipped classroom teaching [21,41]. The result of this study also echoes that flipped classroom can help increases the self-efficacy of nurses in EBN.

### Strengths and limitations

This study has reveals that the flipped classroom can improve nurses’ self-efficacy in EBN. However, there might be a participant bias in our study because of convenient sampling. All participants were enrolled from a medical center in northern Taiwan and the nurses can register the EBN course from internet for free. All participants were assumed to be interested in EBN training. Thus, the convenience sampling might limit the inference of the research results. Besides, studies with a larger sample size can help researchers filter out individual interest bias by adding items to the basic information questionnaire to check participant’s interest in EBN. In addition, the research results were also limit in the knowledge and self-efficacy of EBN, more outcome evaluation approach might be needed for other EBN competences.

### Implications

The application of EBN into clinical has become a focus on quality healthcare, but is often impeded by some factors. It is reported in the literature that lack of EBN skills and organizational support were reasons impeded the implementation of EBN. This study developed a flipped classroom EBN training course conducted by the hospital to support and provide nurses for the EBN skills. The research results showed the supportive program improve the nurses’ EBN knowledge and self-efficacy. The research result implied that flipped classroom EBN training course can be held in hospitals for the enhancement of EBN knowledge and self-efficacy.

## Conclusion and recommendation

This objective of this study was to evaluate the learning effectiveness of the EBN flipped classroom courses on nurses’ knowledge and self-efficacy. The immediate results show the experimental group reported better score in EBN knowledge and self-efficacy than the control group. However, the one-month effect was dropped in both group. The results might due to the 5-hour short-term design of the EBN course. More strategies are recommended for further research to improve the long-term effect of EBN competence.

## Supporting information

S1 File Research raw data. (XLS)
